# Digital Physiotherapeutic Scoliosis-Specific Exercises for Adolescent Idiopathic Scoliosis: A Randomized Clinical Trial

**DOI:** 10.1001/jamanetworkopen.2024.59929

**Published:** 2025-02-18

**Authors:** Wangshu Yuan, Weihong Shi, Lixia Chen, Di Liu, Ye Lin, Qing Li, Jiandong Lu, Houqiang Zhang, Qiyang Feng, Huiling Zhang

**Affiliations:** 1 Department of Rehabilitation Medicine, Peking Union Medical College Hospital, Chinese Academy of Medical Sciences and Peking Union Medical College, Beijing, China; 2 Jiakang Zhongzhi Technology Company, Beijing, China; 3 The University of Chicago, Pritzker School of Medicine, Chicago, Illinois; 4 Tianjin Binhai Vocational Institute of Automotive Engineering, Tianjin, China

## Abstract

**Importance:**

The effectiveness of a training support program of individually tailored physiotherapeutic scoliosis-specific exercises (PSSEs) delivered via a digital care system is unclear.

**Objective:**

To determine the effectiveness of a digital care program in which patients receive PSSE supervision and guidance via the Healbone Intelligent Rehabilitation System and educational videos compared with a conventional intervention involving 3 treatment sessions conducted by qualified physiotherapists at outpatient clinics or specialized orthopedic institutions and unsupervised home-based PSSE training.

**Design, Setting, and Participants:**

This randomized clinical trial recruited patients aged 9 to 17 years from Peking Union Medical College Hospital in Beijing, China, who had adolescent idiopathic scoliosis with a primary curve Cobb angle of 10° or greater and skeletal immaturity (as determined via the Risser grading system). The trial was conducted from June 1, 2023, to August 10, 2024.

**Interventions:**

Patients were randomly assigned to a digital care (DC) group, in which each patient received fully remote and home-based PSSE training supported by a digital care system, or a usual care group, in which each patient completed 3 treatment sessions conducted by qualified physiotherapists at outpatient clinics or specialized orthopedic institutions and home-based PSSE training without supervision.

**Main Outcomes and Measures:**

The primary outcome was a change in the Cobb angle of the major curve between baseline and month 6 of the intervention. Primary and secondary outcomes were evaluated using an independent samples *t* test. The proportion of patients who exhibited disease progression or improvement in the Cobb angle between the 2 groups was compared using a χ^2^ test.

**Results:**

Of 591 patients assessed for eligibility, 436 did not meet the eligibility criteria, 12 declined to participate, and 15 withdrew before randomization. The remaining 128 patients were randomized to 2 parallel groups, with 64 patients in each group (mean [SD] age, 11.1 (2.2) years; 97 female [75.8%]), all of whom completed the baseline assessment and 6-month follow-up. The mean difference in the improvement of the Cobb angle between the DC group and the usual care group after a 6-month intervention was −4.23° (95% CI, −6.08° to −2.39°) in the intention-to-treat analysis and −4.01° (95% CI, −5.68° to −2.35°) in the per-protocol analysis. The posttreatment Cobb angle was adjusted by using analysis of covariance, and the mean of the difference in the adjusted posttreatment Cobb angle between 2 groups was −4.24° (95% CI, −6.09° to −2.38°).

**Conclusions and Relevance:**

In this randomized clinical trial, the effectiveness of individually tailored PSSE training support delivered via a digital care system was superior to that of a conventional PSSE training model in improving the Cobb angle. This mode of exercise may be an effective and convenient alternative for individuals with adolescent idiopathic scoliosis.

**Trial Registration:**

Chinese Clinical Trial Registry Identifier: ChiCTR2300076563

## Key Points

**Question:**

Is digitally supported and home-based physiotherapeutic scoliosis-specific exercise more effective than conventional physiotherapeutic scoliosis-specific exercise for reducing the Cobb angle among children and adolescents with adolescent idiopathic scoliosis?

**Findings:**

In this randomized clinical trial that included 128 patients aged 9 to 17 years who were skeletally immature, digitally supported and home-based physiotherapeutic scoliosis-specific exercise reduced the Cobb angle significantly more than conventional physiotherapeutic scoliosis-specific exercise.

**Meaning:**

The result of this study suggests that digitally supported and home-based physiotherapeutic scoliosis-specific exercise provides a more effective alternative for patients with adolescent idiopathic scoliosis compared with a conventional intervention model.

## Teaser

This randomized clinical trial examines whether digitally supported and home-based physiotherapeutic scoliosis-specific exercise is more effective than conventional physiotherapeutic scoliosis-specific exercise for reducing the Cobb angle among youths with adolescent idiopathic scoliosis.

## Introduction

Adolescent idiopathic scoliosis (AIS) is a complex 3-dimensional spinal deformity of unknown etiology, defined as a spinal curvature of 10° or greater.^1,2^ According to scoliosis-screening studies conducted between 1985 and 2011, the prevalence of AIS is estimated to be between 0.5% and 5.2%.^3,4^ Among adolescents with a Cobb angle greater than 20°, the likelihood of disease progression is 70% or more.^5,6^ According to the recommendations of the International Society on Scoliosis Orthopaedic and Rehabilitation Treatment (SOSORT),^7,8^ conservative treatment should be provided to individuals with AIS to prevent the progression of scoliosis before skeletal maturity is reached.

Physiotherapeutic scoliosis-specific exercises (PSSEs) are among the most crucial conservative treatments for patients with AIS.^7,8,9,10^ However, compared with conventional exercises such as resistance training, PSSE requires a deeper understanding by patients of the specific exercises tailored to their type of scoliosis, making mastery more complex and challenging for patients with AIS. Additionally, patients with AIS must adhere consistently to a standardized PSSE regimen over the long term and develop habits of daily self-management to ensure the efficacy of PSSE.^11,12^ In previous high-intensity evidence-based trials,^10,13,14,15^ the traditional PSSE model involved an initial assessment and 1 to 3 treatment sessions conducted by qualified physiotherapists at outpatient clinics or specialized orthopedic institutions, after which patients completed the PSSE regimen at home without supervision.

However, the traditional PSSE model faces several challenges.^16,17,18,19,20^ On the one hand, due to the scarcity of medical resources (eg, specialized therapists and facilities) and limitations in time restriction, the high expense of traveling and treatment, and the transportation inconvenience, a significant proportion of individuals with AIS are unable to access professional guidance.^16,17,18^ On the other hand, due to a lack of awareness of the importance of home-based self-efficacy, patients with AIS often fail to complete their home-based self-efficacy programs and do not develop habits of daily self-management.^19,20^ Digital interventions hold significant potential in overcoming these challenges.^21^ However, few studies have explored the effectiveness of digital PSSE programs and daily posture management for patients with AIS.^11^

The Healbone Intelligent Rehabilitation System (HIRS) is a user-centered smartphone application that integrates remote supervision and guidance of PSSE training with scoliosis-related educational videos and articles to complete a long-term PSSE program and daily self-management. The purpose of this randomized clinical trial was to compare the treatment outcomes of a digital care (DC) group receiving PSSE supervision and guidance through the HIRS and educational videos with those of a usual care group following the traditional PSSE model.

## Methods

### Study Design, Setting, and Participants

This was a single-center, parallel-group randomized clinical trial conducted from June 1, 2023, to August 10, 2024, in accordance with the requirements of the Declaration of Helsinki.^22^ The study received approval from the ethics committee of Peking Union Medical College Hospital (PUMCH). Written informed consent was obtained from all participants. The trial protocol is provided in Supplement 1. The study followed the Consolidated Standards of Reporting Trials (CONSORT) reporting guideline.

All participants were patients recruited from the Department of Rehabilitation Medicine at PUMCH. Two physicians (L.C. and Q.L.) screened patients to determine their eligibility based on the inclusion criteria and observed or followed them up until the conclusion of the study. Prior to the trial, all patients were informed that no interventions other than PSSE training were permitted. If a physiotherapist (W.Y. and W.S.) noticed that a patient had not used the HIRS for an extended period, they would promptly inquire about reasons for the patient’s failure to engage in PSSE training. After obtaining informed consent, patients were enrolled in the study. The inclusion criteria were untreated child and adolescent males and females with AIS; having a primary curve Cobb angle of 10° or greater; being aged 9 to 17 years; having a Risser grade ranging from 0 to 4 (on a scale of 0-5, where higher grades indicate greater skeletal maturity), with female children and adolescents being within 1 year after menarche^23^; being capable of understanding and completing complex motor tasks; and patients or their parents being proficient in using a smartphone. The exclusion criteria were having a nonidiopathic etiology of scoliosis determined by clinical information, physical examination, or medical imaging; having other spinal disorders (eg, tumors); or having limb-length discrepancy. Before participating in the trial, 2 experienced physicians (L.C. and Q.L.) assessed whether patients met the inclusion criteria through imaging evaluation. Patients were excluded if they withdrew from the study or failed to engage in any exercise regimen for 28 consecutive days.

This study strictly adhered to the PUMCH-SSE classification for all patients with AIS. The PUMCH-SSE classification,^24^ based on the PUMC system,^25,26^ was established to guide PSSE training for patients with AIS. The PUMCH-SSE classification is divided into 9 subtypes, ranging from type 0 (slight curve) to type III (distinguished based on the number of apexes) (eTable 1 in Supplement 2). Patients with a Cobb angle less than 10° were not included in this study and thus were classified as PUMCH-SSE type 0.^24^

### Randomization

Patients were randomly allocated to either the DC group or the usual care group using an online platform for randomization.^27^ Subsequently, patients in the DC group were assigned the letter D, while those in the usual care group were assigned the letter C. Based on the results generated by the online platform (eg, C, D, C, D, …), slips of paper labeled with the letters D and C were placed in sealed, opaque, and uniformly sized envelopes. After all baseline measurements were completed for all patients, the envelopes were sequentially opened to reveal the allocation results. The allocation sequence was prepared by an independent researcher, not involved in the study, using simple randomization.

### Intervention and Control Conditions

In this trial, the HIRS was provided free of charge to patients with AIS in the DC group, and physiotherapists (W.Y. and W.S.) encouraged timely use of the HIRS using a smartphone. In the market, the expense of purchasing the HIRS is lower than that of receiving PSSE guidance at outpatient clinics or specialized orthopedic institutions. The HIRS consists of 3 distinct components: the physician’s interface, the user’s interface, and the data-storage module, as depicted in the eFigure in Supplement 2. Our research team involved 2 senior physiotherapists (W.Y. and W.S.), each with over 5 years’ experience in the diagnosis and conservative management of scoliosis. One of the therapists (W.Y.) is a member of the SOSORT.

Prior to the commencement of the trial, experienced physiotherapists (W.Y. and W.S.) and orthopedic surgeons cocreated specific educational videos related to scoliosis, along with detailed instructions, which were uploaded to the HIRS. The video is divided into 6 topics, titled The Unignorable Issue of Adolescent Scoliosis, Screening Methods for Scoliosis, Postural Management in Daily Life (including sitting, standing, walking, and lying down), Daily PSSE Training, Treatment Approaches for Scoliosis of Varying Severity, and Common Issues in the Daily Activities of Patients With Scoliosis. Individuals in the DC group were required to regularly view educational videos related to scoliosis to enhance their understanding of the condition.^28^ In this trial, individuals in the DC group received a single in-person PSSE training session under the guidance of physiotherapists in an outpatient setting. The HIRS was installed on the smartphones of patients in the DC group, and personal accounts were registered using the application. Patients were required to access the application using their personal accounts each time they engaged in PSSE training. For the supervision of the DC group, the HIRS supervises home-based PSSE training of patients with AIS involving the following steps. First, under the digital PSSE model, the HIRS regularly monitors the completion status of home-based PSSE training for patients with AIS. If patients fail to complete their training, the HIRS automatically sends reminders through the chat window to prompt timely completion. Second, if patients continue to miss their PSSE training over a period, the HIRS notifies the physiotherapist, who then contacts the patient to ensure training compliance. Finally, if patients experience difficulties or physical discomfort, the physiotherapist can reach out to a physician for medical support.

The PSSE training consisted of daily self-correction exercises and overcorrection exercises. The daily self-correction exercises were based on the Scientific Exercise Approach to Scoliosis method,^7,8^ in which therapists instruct patients with AIS in specific 3-dimensional corrective postures. Physiotherapists required patients with AIS to integrate the self-corrective position into routine activities such as sitting, standing, and walking whenever possible. If patients were unable to actively control their posture continuously, they were encouraged to use aids, such as insoles or cushions, to achieve passive correction. Moreover, considering that all patients were students who spent the majority of their day sitting for academic activities, physiotherapists further instructed all patients with AIS to perform at least 30 minutes of seated strengthening exercises daily while maintaining a self-corrected posture. The overcorrection exercises were derived from the Schroth method.^11,29,30^ The PSSE training regimen, established based on the PUMCH-SSE classification, has been demonstrated to be effective for patients with AIS.^24^ All patients with AIS engaged in overcorrection exercises for 30 minutes per day, at least 5 days each week, for a duration of 6 months. The PSSE training programs were not progressive, and the HIRS was shown repeatedly to patients in the DC group in the form of videos. The HIRS automatically recorded individual PSSE training performance and engagement data, including the number of sessions and duration of training.

In the usual care group, individuals received three 90-minute in-person training sessions per month, under the guidance of physiotherapists, during the first 3 months, with additional guidance sessions provided as needed. For the supervision of the usual care group, physiotherapists instructed patients or their parents to record the number of days of self-correction exercise per week and the duration of overcorrection exercise each time, which may facilitate self-monitoring and parental supervision.

### Data Collection

All patients underwent baseline measurements at the Department of Rehabilitation, PUMCH. After a 6-month intervention, all patients were required to return to the hospital for assessment. The primary outcome measure was the change in the Cobb angle of the primary curve between baseline and the 6-month follow-up. The Cobb angle was measured on standing radiographs by 2 physiotherapists who determined the angle between the most tilted vertebrae at the upper and lower ends of the curve. The final Cobb angle was the average of the 2 measurements. Receiver operating characteristic curve analysis was used to determine the minimal clinically important difference for improvement in the Cobb angle after a 6-month PSSE intervention, which was identified as 3.50°.^31,32^ Charalampidis et al^10^ defined treatment success in patients with moderate AIS as no progression in scoliosis in individuals with a Risser grade 4 or less after a 6-month PSSE intervention.

The research team defined a reduction in the primary curve Cobb angle of 0° to 5° as mild improvement, a reduction of more than 6° as substantial improvement, and an increase in the primary curve Cobb angle as progression. Our research team evaluated the improvement in vertebral rotation at the spinal level in patients with AIS based on the angle of trunk rotation improvement after the 6-month intervention, and the patients’ angle of trunk rotation was measured by a scoliometer (Orthopedic Systems Inc).

Pelvic coronal plane asymmetry negatively affects the gait of patients with AIS.^33^ The pelvic tilt angle during the gait cycle was measured using the Movit Gait system (Sensor Medica S.r.l.). Four representative time points within the gait cycle were selected for analysis, corresponding to the minimum and maximum values during the stance phase and the minimum and maximum values during the swing phase. The validity and reliability of the Movit Gait system have been established.^34,35^

The assessment of engagement was conducted through the following metrics: (1) adherence to home-based exercise performance, including the frequency of overcorrective exercises per week, the duration of overcorrective exercises per week, and the number of individuals in both groups who consistently practiced self-correction exercises daily, and (2) dropout rates. Data for the DC group was automatically collected by the HIRS, while usual care group data was manually recorded by individuals or their patients.

### Statistical Analysis

The sample size was calculated using PASS, version 11 (NCSS Statistical Software). In accordance with the principles of clinical superiority trials and preliminary trial results, the mean difference (SD) in the improvement of the Cobb angle between the DC group and the usual care group was 6 (4) and a superiority margin of 4 for the Cobb angle. Assuming a power of 80% and a 2-sided significance level of *P* = .05, the estimated sample size would have been 102 patients (51 per group). Accounting for a 20% dropout rate, a total of 128 patients (64 per group) would have been needed to be enrolled.

Demographic data are presented as mean (SD) values for continuous variables and as numbers (percentages) for categorical variables. The primary and secondary outcomes of this study are reported as mean (SD) values and 95% CIs. The distribution of continuous variables was assessed using the Kolmogorov-Smirnov test, followed by verification through histograms and Q-Q plots. Given the assumption of normality, primary and secondary outcomes were evaluated using the independent samples *t* test. The proportion of patients who exhibited disease progression or improvement in the Cobb angle between the 2 groups was compared using the χ^2^ test. Both intention-to-treat and per-protocol analyses were conducted for outcome assessment. The posttreatment outcomes were evaluated using analysis of covariance to adjust for baseline differences between the groups. Missing data were handled using multiple imputation by chained equations.^36^

All analyses were conducted under a 2-tailed hypothesis with an α significance level of .05. Statistical analyses were performed using SPSS Statistics, version 23 (IBM Inc) and RStudio package in R, version 4.3.1 (R Project for Statistical Computing) and were performed by a blinded statistician (Houqiang Zhang).

## Results

### Patient Characteristics

**Figure 1.**
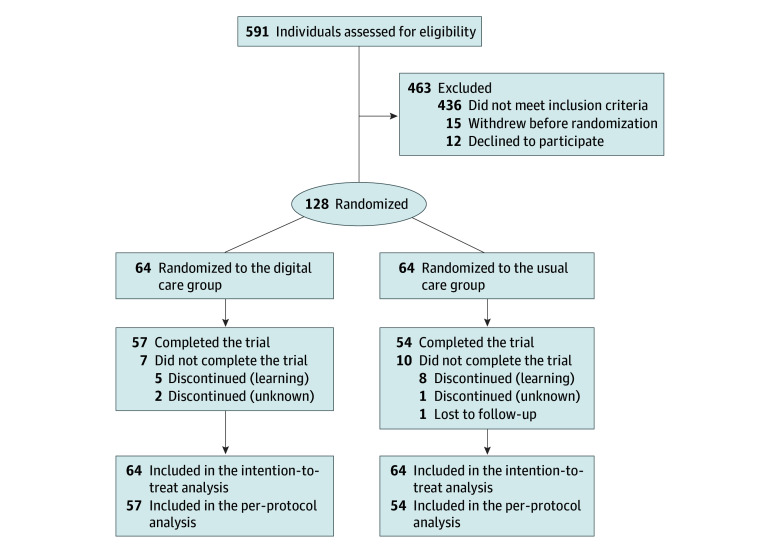
Patient Flow Diagram of Trial Participation

A total of 591 patients underwent eligibility screening: 436 did not meet the inclusion criteria, 12 declined participation, and 15 withdrew before randomization. Ultimately, 128 patients (mean [SD] age, 11.1 [2.2] years; 97 female [75.8%] and 31 male [24.2%]), who completed the baseline assessment and 6-month follow-up, were randomly and evenly assigned to either the DC group (n = 64) or the usual care group (n = 64). The completion rate of the follow-up and the PSSE training program was 89.1% (57 of 64) in the DC group and 84.4% (54 of 64) in the usual care group, as depicted in Figure 1. The clinical and demographic characteristics of patients in both groups were comparable (Table 1).

**Table 1.** Baseline Characteristics of Study Patients

Characteristic	Patient group	
	Digital care (n = 64)	Usual care (n = 64)
Age, mean (SD), y	10.95 (2.13)	11.31 (2.33)
Sex, No. (%)		
Female	49 (76.6)	48 (75.0)
Male	15 (23.4)	16 (25.0)
Height, mean (SD), cm	150.34 (14.93)	151.84 (13.53)
Weight, mean (SD), kg	38.41 (11.09)	39.77 (10.79)
BMI, mean (SD)	16.82 (2.14)	16.85 (2.21)
AIS grade, No. (%)^a^		
Mild	50 (78.1)	47 (73.4)
Moderate	14 (21.9)	17 (26.6)
Risser grade, No. (%)^b^		
0	33 (51.6)	21 (32.8)
1	4 (6.3)	11 (17.2)
2	8 (12.5)	14 (21.8)
3	10 (15.5)	9 (14.1)
4	9 (14.1)	9 (14.1)
5	NA	NA
PUMCH-SSE classification for patients with AIS, No. (%)^c^		
0	NA	NA
I0	1 (1.6)	2 (3.1)
Ia	4 (6.3)	1 (1.6)
Ib	24 (37.5)	28 (43.7)
Ic	15 (23.4)	13 (20.3)
IIa	2 (3.1)	2 (3.1)
IIb	15 (23.4)	16 (25)
IIc	2 (3.1)	1 (1.6)
III	1 (1.6)	1 (1.6)

Abbreviations: AIS, adolescent idiopathic scoliosis; BMI, body mass index (calculated as weight in kilograms divided by height in meters squared); NA, not applicable; PUMCH-SSE, Peking Union Medical College Hospital scoliosis-specific exercise.

^a^ A primary Cobb angle of 10° to 20° was defined as mild scoliosis; a maximum Cobb angle of 20° to 40° was defined as moderate scoliosis.

^b^ Scores range from 0 to 5, with higher grades indicating greater skeletal maturity.^23^ Patients with Risser grade 5 were excluded from this study.

^c^ Types range from type 0 (slight curve) to type III (distinguished based on the number of apexes). Patients classified as type 0 by the PUMCH-SSE classification, due to a Cobb angle less than 10°, were not included in this study.^24^ The Fisher exact test was used to analyze the distribution of different types of patients with AIS, according to the PUMCH-SSE classification system.

### Patient Engagement

The mean (SD) frequency (5.30 [0.57] times vs 4.63 [0.60] times; *P* < .001) and duration of overcorrection exercises (179.44 [24.18] minutes vs 147.84 [23.75] minutes; *P* < .001) per week were significantly higher in the DC group compared with the usual care group. Furthermore, a greater proportion of patients in the DC group adhered to daily self-correction exercises for more than 6 days per week compared with the usual care group (52 [91.2%] vs 36 [66.7%]; *P* = .003) (Table 2).

**Table 2.** Engagement Metrics of Patients

Engagement variable	Patient group		*P* value
	Digital care^a^	Usual care^b^	
Frequency of overcorrection exercises, mean (SD), times/wk			
ITT analysis	5.07 (0.87)	4.40 (0.79)	<.001
PP analysis	5.30 (0.57)	4.63 (0.60)	<.001
Duration of overcorrection exercises, mean (SD), min/wk			
ITT analysis	172.29 (32.60)	139.22 (31.36)	<.001
PP analysis	179.44 (24.18)	147.84 (23.75)	<.001
Individuals who persisted in daily self-correction exercises over 6 d/wk, No. (%)			
ITT analysis	55 (85.9)	40 (62.5)	.001
PP analysis	52 (91.2)	36 (66.7)	.003

Abbreviations: ITT, intention-to-treat; PP, per-protocol.

^a^ There were 64 patients in the ITT analysis and 57 patients in the PP analysis.

^b^ There were 64 patients in the ITT analysis and 54 patients in the PP analysis.

### Primary Outcome

**Figure 2.**
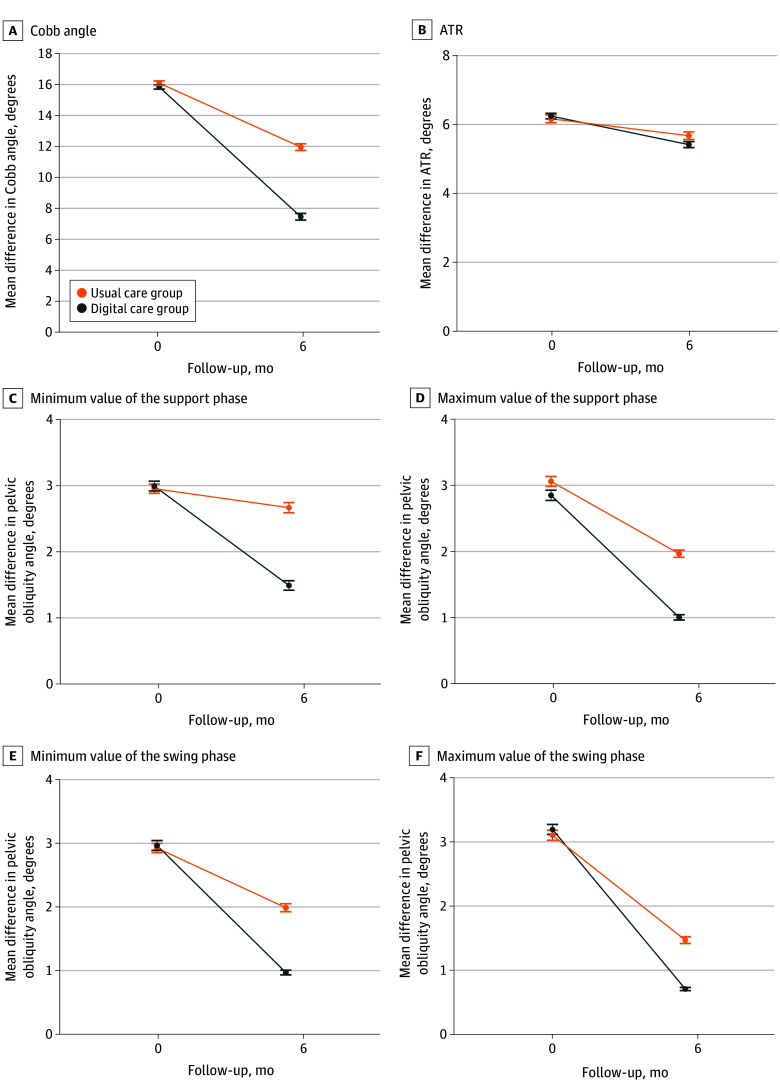
Improvement of Primary and Secondary Outcomes A, Primary outcome. B-F, Secondary outcomes. Improvement of the pelvic obliquity angle in 4 representative time points of the gait cycle is shown in panels C through F. Error bars represent 95% CIs. ATR indicates angle of trunk rotation.

The results of the intention-to-treat analysis (Table 3 and Figure 2) were consistent with the per-protocol analysis (eTable 2 in Supplement 2) in the primary and secondary outcomes. The adjusted posttreatment primary and secondary outcomes are shown in eTable 3 in Supplement 2.

**Table 3.** Intention-to-Treat Analysis Outcome Changes From Baseline to 6 Months^a^

Outcome variable	Patient group		Mean difference between groups	*P* value
	Digital care (n = 64)	Usual care (n = 64)		
Cobb angle of the major curve				
Baseline	15.78 (14.70 to 16.86)	16.03 (14.90 to 17.16)	−0.25 (−1.80 to 1.30)	NA
6 mo	7.42 (5.71 to 9.14)	11.91 (10.19 to 13.63)	−4.48 (−6.89 to −2.08)	<.001
Changes from baseline to 6 mo	−8.36 (−9.73 to −6.99)	−4.13 (−5.39 to −2.86)	−4.23 (−6.08 to −2.39)	<.001
Progression and improvement of the Cobb angle of the major curve in patients with AIS, No. (%)				
Large improvement	49 (76.6)	29 (45.3)	NA	.001
Mild improvement	7 (10.9)	21 (32.8)	NA	
Progression	8 (12.5)	14 (21.9)	NA	
Angle of trunk rotation, degree				
Baseline	6.26 (5.63 to 6.90)	6.15 (5.24 to 7.05)	0.12 (−0.98 to 1.21)	NA
6 mo	5.40 (4.70 to 6.09)	5.66 (4.78 to 6.54)	−0.26 (−1.37 to 0.85)	.83
Changes from baseline to 6 mo	−0.87 (−1.30 to −0.43)	−0.49 (−0.94 to −0.04)	−0.38 (−1.00 to 0.24) ·	.64
Pelvic obliquity angle				
Minimum value of the support phase				
Baseline	3.03 (2.44 to 3.62)	2.99 (2.42 to 3.55)	0.04 (−0.77 to 0.85)	NA
6 mo	1.51 (1.10 to 1.92)	2.70 (2.07 to 3.33)	−1.19 (−0.94 to −0.45)	.002
Changes from baseline to 6 mo	−1.52 (−2.21 to −0.84)	−0.29 (−1.08 to 0.05)	−1.23 (−2.27 to −0.19)	.02
Maximum value of the support phase				
Baseline	2.84 (2.22 to 3.46)	3.05 (2.46 to 3.96)	−0.21 (−1.06 to 0.64)	NA
6 mo	1.00 (0.69 to 1.31)	1.97 (1.52 to 2.41)	−0.97 (−1.50 to −0.43)	<.001
Changes from baseline to 6 mo	−1.85 (−2.38 to −1.31)	−1.08 (−1.48 to −0.69)	−0.76 (−1.42 to −0.10)	.02
Minimum value of the swing phase				
Baseline	2.97 (2.36 to 3.59)	2.93 (2.36 to 3.50)	0.04 (−0.79 to 0.87)	NA
6 mo	0.97 (0.66 to 1.27)	1.99 (1.49 to 2.49)	−1.02 (−1.60 to −0.44)	.001
Changes from baseline to 6 mo	−2.01 (−2.55 to −1.47)	−0.94 (−1.60 to −0.24)	−1.06 (−1.90 to −0.22)	.02
Maximum value of the swing phase				
Baseline	3.18 (2.56 to 3.80)	3.09 (2.47 to 3.72)	0.08 (−0.79 to 0.96)	NA
6 mo	0.70 (0.50 to 0.90)	1.46 (1.04 to 1.88)	−0.76 (−1.22 to −0.30)	.004
Changes from baseline to 6 mo	−2.47 (−3.06 to −1.89)	−1.63 (−2.22 to −1.04)	−0.84 (−1.67 to −0.02)	.04

Abbreviations: AIS, adolescent idiopathic scoliosis; NA, not applicable.

^a^ Data are presented as mean (95% CI), degree unless otherwise indicated.

The baseline Cobb angle of the major curve was similar between the 2 groups (mean difference, −0.25° [95% CI, −1.80° to 1.30°]; *P* = .08), and both groups showed improvement after 6 months of intervention (DC group: −8.36° [95% CI, −9.73° to −6.99°] and usual care group: −4.13° [95% CI, −5.39° to −2.86°]; both *P* < .001). After 6 months of intervention, both the DC group and the usual care group exhibited clinically significant improvements in the Cobb angle exceeding 3.5°. The degree of improvement in the Cobb angle after 6 months was significantly greater in the DC group compared with the usual care group (intention-to-treat analysis: −4.23° [95% CI, −6.08° to −2.39°]; *P* < .001 and per-protocol analysis: −4.01° [95% CI, 5.68° to −2.35°]; *P* < .001). In addition, the mean of the difference of the adjusted posttreatment Cobb angle between the 2 groups was −4.24° (95% CI, −6.09° to −2.38°; *P* < .001). In summary, after the 6-month intervention, the mean improvement in the Cobb angle for both groups exceeded the superiority margin of 4.0°.

The proportion of patients with significant Cobb angle improvement was higher in the DC group (49 of 64 [76.6%]) than in the usual care group (29 of 64 [45.3%]). There was a statistically significant difference between the DC group and the usual care group (*P* = .001) (Table 3).

### Secondary Outcomes

After the 6-month intervention, the difference in the angle of trunk rotation improvement between the 2 groups was not statistically significant (mean, −0.38° [95% CI, −1.00° to 0.24°]; *P* = .64) (Table 3). In addition, the improvement in pelvic tilt angles at the 4 representative time points in the gait cycle, including the minimum value during the support phase (mean difference, −1.23° [95% CI, −2.27° to −0.19°]; *P* = .02), the maximum value during the support phase (mean difference, −0.76° [95% CI, −1.42° to −0.10°]; *P* = .02), the minimum value during the swing phase (mean difference, −1.06° [95% CI, −1.90° to −0.22°]; *P* = .02), and the maximum swing phase value (mean difference, −0.84° [95% CI, −1.67° to −0.02°]; *P* = .04), was significantly greater in the DC group than in the usual care group (Table 3).

## Discussion

The objective of this randomized clinical trial was to evaluate the efficacy of the home-based PSSE program for patients with AIS using the HIRS in comparison with the conventional PSSE approach. In this trial, both intervention groups demonstrated high levels of engagement, with a low dropout rate. Before the 6-month PSSE intervention, the baseline characteristics of patients in both groups were comparable. For the primary outcome measure, the posttreatment Cobb angle was adjusted using the intention-to-treat analysis, the per-protocol analysis, and analysis of covariance. All analyses consistently demonstrated that patients with AIS in the DC group exhibited significantly greater improvement in the Cobb angle after the 6-month intervention compared with the usual care group, with statistically significant differences. Furthermore, the mean difference in Cobb angle improvement between the 2 groups exceeded 4.0°. These findings indicate that the digital PSSE model achieved significantly better than did the traditional PSSE model in improving the Cobb angle for patients with AIS. Although both groups showed improvements in pelvic coronal tilt during walking and angles of trunk rotation after the 6-month intervention, further investigation is needed to fully evaluate the efficacy of the digital PSSE training approach. Additionally, the bidirectional communication established through the HIRS played a positive role in promoting the outcomes of the trial by promptly addressing any questions or concerns raised by patients in the DC group.

Conservative treatment for AIS, including PSSEs and bracing, aims to prevent the progression of scoliosis while correcting spinal and trunk deformities over the long term and to improve health-related quality of life while preventing deterioration.^37,38,39^ However, due to limited medical resources as well as geographic, time, and financial constraints, mastering the PSSE regimen under the traditional PSSE model can be challenging for patients with AIS.^39,40,41^ PSSE training, supported by a digital care system, may be an effective approach to address the current challenges. Manzak Dursun et al^17^ enrolled 31 patients with AIS and randomly assigned them to an experimental group (n = 16) and a control group (n = 15). The experimental group performed scoliosis-specific Pilates training using a hybrid remote rehabilitation approach, while the control group completed the same training through a home-based rehabilitation method. The results of the trial showed that after 12 weeks of intervention, the improvement in the Cobb angle was significantly greater in the experimental group compared with the control group. However, the small sample size in this study limited its validity and generalizability. In this context, the randomized clinical trial in this study provides evidence for the effectiveness of a fully remote PSSE training program based on a digital care plan, which addresses the shortcomings of the traditional PSSE model.^16,17,42^ The PSSE model, centered around digital health care, laid the foundation for the development of an integrated, multidisciplinary health care model that combines digital and face-to-face approaches, with profound clinical, cultural, and organizational implications for health care practitioners.^43^

### Strengths and Limitations

The strengths of this study lie in its methodological rigor—a randomized clinical trial comparing digital system-supported home-based PSSE training with the traditional PSSE model. It is noteworthy that both groups adhered to guideline-recommended treatment dosages.^7,8^ The novelty of the digital intervention lies in the development of fully remote PSSE training and scoliosis educational videos under the support of the HIRS, along with the establishment of bidirectional communication. Compared with previous studies, this research included a larger sample size of patients with AIS. However, as patients with AIS may continue to experience progression in their Cobb angle prior to skeletal maturity, long-term intervention and follow-up are essential. In the future, we plan to conduct multicenter randomized clinical trials with extended intervention and follow-up periods to further validate the efficacy of the digital PSSE training model.

The study also has limitations. The PUMCH-SSE classification system used in this study is more accurate and systematic compared with other classification methods, as each category within the PUMCH-SSE classification corresponds to a specific PSSE training protocol.^25,26^ However, the incidence of patients classified as types I0, IIa, IIc, and III within the PUMCH-SSE system was relatively low, resulting in a limited number of patients with AIS of these types among the overall study population. Additionally, the proportion of patients with moderate scoliosis was lower than that of patients with mild scoliosis, primarily because patients with moderate scoliosis tend to prefer combined PSSE training and brace treatment, which was not the focus of this trial.

## Conclusion

In this randomized clinical trial, a total of 128 patients with AIS were enrolled. The results show that fully remote and home-based PSSE training support delivered using a digital care system was superior to the traditional PSSE model in improving the Cobb angle. This mode of exercise may provide a more effective and convenient alternative for individuals with AIS.

## Notes

Supplement 1.
Trial Protocol

Supplement 2. **eTable 1.** PUMCH-SSE Classification **eTable 2.** Changes in Outcomes From Baseline to 6 Months: Per-Protocol Analysis **eTable 3.** Adjusted Primary and Secondary Posttreatment Outcomes **eFigure.** Example of the HIRS

Supplement 3.
Nonauthor Collaborators

Supplement 4.
Data Sharing Statement
