# Supplementary material for: Digital Physiotherapeutic Scoliosis-Specific Exercises for Adolescent Idiopathic Scoliosis: A Randomized Clinical Trial
Source: JAMA Netw Open. 2025 Feb 18;8(2):e2459929. doi: 10.1001/jamanetworkopen.2024.59929 (PMC11836762; doi:10.1001/jamanetworkopen.2024.59929)
Supplement: Supplement 1. — Trial Protocol [file jamanetwopen-e2459929-s001.pdf]

Effect of the 6-month digital-based physiotherapeutic specific scoliosis exercise for patients with Adolescent idiopathic scoliosis: A Randomized Controlled Trial

Study protocol

Peking Union Medical College Hospital, Chinese Academy of Medical Sciences and Peking Union Medical College, Beijing, China

Version： 1.0

DATE: 2022-11-30

Catalogue

1.Background **3**
2.Reseach Objective **4**
3.Reseach Method **4**
3.2 Secondary Outcome： 5
3.3 The definition of outcomes 5
4.Data analysis and the calculation of sample size **6**
4.1 Null hypothesis: 6
4.2 Alternative hypothesis: 7
4.3 Statistical Method 7
4.4 The calculation of sample size 9
4.5 The method of Allocation 9
5.Exclusion and Inclusion ceriteria **9**
5.1 Inclusion criteria 9
5.2 Exculsion criteria： 10
6.Research process **10**
6.1 The patients’ baseline assessment 10
6.2 Allocation 10
6.3 Intervention 10
7. The follow-up **13**
8. The Figure of research proccess **14**
9. Reference **14**

1.Background

Adolescent idiopathic scoliosis (AIS) is a complex three-dimensional spinal deformity of unknown etiology, defined as a spinal curvature of ≥10°[1, 2]. According to scoliosis screening studies conducted between 1985 and 2011, the prevalence of AIS is estimated to be between 0.5% and 5.2%[3, 4]. It is generally accepted that the e incidence of AIS among individuals under the age of 16 is approximately 2% to 3%[3, 4]. Among adolescents with a Cobb angle greater than 20°, the likelihood of disease progression is 70% or more[5, 6]. According to the recommendations of the International Society on Scoliosis Orthopaedic and Rehabilitation Treatment (SOSORT)[7, 8], conservative treatment should be provided to adolescents with AIS to o prevent the progression of scoliosis before skeletal maturity is reached.

Physiotherapy Scoliosis-Specific Exercises (PSSE) are among the most crucial conservative treatments for patients with AIS[7, 8]. Moderate-certainty evidence from m randomized controlled trials (RCTs) supports the effectiveness of PSSE in slowing the progression of scoliosis in AIS patients and even reducing the degree of curvature[9, 10]. However, compared to conventional exercises such as resistance e training, PSSE requires a deeper understanding by patients of the specific exercises tailored to their type of scoliosis, making mastery more complex and challenging for AIS patients. Additionally, patients with AIS must adhere consistently to a standardized PSSE regimen over the long term and develop habits of daily self-management to ensure the efficacy of PSSE[11, 12]. In previous high-intensity y evidence-based trials[10, 13, 14, 15], the traditional PSSE model involved an initial assessment and 1 to 3 treatment sessions conducted by qualified physiotherapists at outpatient clinics or specialized orthopedic institutions, after which patients completed the PSSE regimen at home without supervision.

However, the traditional PSSE model faces several challenges. On one hand, due to the scarcity of medical resources—including specialized therapists and facilities—as well as limitations in time, cost, and transportation, a significant proportion of adolescents with AIS are unable to access professional guidance[16-18]. On the other r hand, due to a lack of awareness of the importance of home-based self-efficacy, AIS patients often fail to complete their home-based self-efficacy programs and do

not develop habits of daily self-management, resulting in sub-optimal outcomes in home-based self-efficacy training[19, 20].

Digital interventions hold significant potential in overcoming these challenges, enhancing individual adherence and empowerment, and aiding individuals in managing their daily posture and completing PSSE training programs in a timely manner[21]. However, few studies have explored the effectiveness of digital interventions in PSSE programs and daily posture management for AIS patients.

The Healbone Intelligent Rehabilitation System (HIRS) is a user-centered smartphone application that integrates remote supervision and guidance of PSSE training with scoliosis-related educational videos and articles. In our previous work, we demonstrated the efficacy of the HIRS system in patients with nonspecific low back pain[22]. The purpose of this RCT is to compare the treatment outcomes of a Digital Care Group (DCG) receiving PSSE supervision and guidance through the HIRS system (Beijing, CN) and educational videos with those of a Conventional Intervention Group (CIG) following the traditional PSSE model.

2.Research Objective

The Healbone Intelligent Rehabilitation System (HIRS) is a user-centered smartphone application that integrates remote supervision and guidance of PSSE training with scoliosis-related educational videos and articles. In our previous work, we demonstrated the efficacy of the HIRS system in patients with nonspecific low back pain[22]. The purpose of this RCT is to compare the treatment outcomes of a Digital Care Group (DCG) receiving PSSE supervision and guidance through the HIRS system (Beijing, CN) and educational videos with those of a Conventional Intervention Group (CIG) following the traditional PSSE model.

3.Reseach Method

This study is a single-center, parallel-group, randomized controlled trial conducted in accordance with the requirements of the Declaration of Helsinki. The study adheres to the CONSORT guidelines

3.1 Primary Outcome： The change of the Cobb angle

3.2 Secondary Outcome：

(1) The change of the angle of trunk rotation, ATR；
(2) The changes in pelvic tilt angle during the gait cycle between baseline and the 6-month follow-up.
(3) Engagement metrics of participants.
(4) The population of AIS patients with progression and improvement of Cobb angle of the major curve

3.3 The definition of outcomes

All subjects will undergo baseline measurements at the Department of Rehabilitation, Peking Union Medical College Hospital. After a 6-month intervention, all participants are required to return to the hospital for a 6-month follow-up assessment. The primary outcome measure is the change in the Cobb angle of the primary curve between baseline and the 6-month follow-up. The Cobb angle will be measured on standing radiographs by two physical therapists (PTs) who will determine the angle between the most tilted vertebrae at the upper and lower ends of the curve. The final Cobb angle will be the average of the two measurements. Secondary outcomes are listed in Table 5.

Study secondary outcomes (clinical and engagement)

Outcome measure	Description
The population of AIS patients with progression and improvement of Cobb angle of the major curve	The research team defines a reduction in the primary curve Cobb angle of 0° to 5° as "mild improvement," a reduction of more than 6° as "substantial improvement," and an increase in the primary curve Cobb angle as "progression." This study analyzed the populations of AIS patients in the DCG and CIG groups in terms of progression, "mild improvement," and "substantial improvement."
The improvement in the angle of trunk rotation (ATR)	To evaluate the improvement in vertebral rotation at the spinal level in patients with AIS (Idiopathic Scoliosis) based on ATR (Angle of Trunk Rotation) improvement at baseline and 6-month follow-up, the following procedure is conducted:

	The subject is asked to stand with feet shoulder-width apart, and the examiner is seated behind the subject. The subject is instructed to slowly bend forward until the spinal deformity is most prominent, with the apex of the hump aligned with the examiner’s line of sight. A scoliometer (Orthopedic Systems INC, Union City, California, USA) is placed on the spinous process at the apex of the curve, and the ATR angle is recorded.
The changes in pelvic tilt angle during the gait cycle between baseline and the 6-month follow-up.	Pelvic coronal plane asymmetry negatively impacts the gait of patients with AIS. The pelvic tilt angle during the gait cycle is measured using the MOVIT Gait System (Sensor Medica s.r.l., Via Bruno Pontecorvo, 1300012 Guidonia Montecelio (RM), Italy). Changes in this angle from baseline to the 6-month follow-up are utilized to assess improvements in pelvic tilt. Four representative time points within the gait cycle are selected for analysis, corresponding to the minimum and maximum values during the stance phase, and the minimum and maximum values during the swing phase. The validity and reliability of the MOVIT Gait System have been established[24,25].
Engagement	The assessment of engagement will be conducted through the following metrics: (i) adherence to home-based exercise performance, including the frequency of over-corrective exercises per week, the duration of over-corrective exercises per week, and the number of individuals in both groups who consistently practice self-correction exercises daily; (ii) dropout rates. Data for the DCG will be automatically collected by the HIRS system, while CIG data will be manually recorded by individuals or their patients.

4.Data analysis and the calculation of sample size

4.1 Null hypothesis: The improvement of Cobb Angle of AIS patients in the Digital Care group (DCG) is less than or equal to the improvement of Cobb Angle of AIS patients in the Conventional Intervention group (CIG);

4.2 Alternative hypothesis: The improvement of Cobb Angle of AIS patients in the DCG group is better than that of AIS patients in the CIG group

4.3 Statistical Method

The distribution of continuous variables was assessed using the Kolmogorov-Smirnov test, followed by verification through histograms and Q-Q plots. Demographic data were presented as mean (standard deviation) for continuous variables and as number (percentage) for categorical variables. Baseline demographic characteristics and engagement metrics between groups were compared using independent samples t-test or Mann-Whitney U test for quantitative variables, and Chi-square test or Fisher's exact test for categorical variables.

The effects of each intervention on primary and secondary outcomes were evaluated based on the 6-month endpoint outcomes and the changes between baseline and 6 months. Both intention-to-treat (ITT) and per-protocol analyses were conducted for outcome assessment. Missing data were handled using multiple imputation by chained equations (MICE)[26].

Receiver operating characteristic (ROC) curve analysis was employed to determine the minimal clinically important difference (MCID) for improvement in Cobb angle post-PSSE intervention at 6 months, which was identified as 3.50 degrees[27,28]. However, Charalampidis A et al.[10] defined treatment success in patients with moderate idiopathic scoliosis as no progression in scoliosis in individuals with a Risser grade ≤4 after 6 months of PSSE intervention.

The primary and secondary outcomes of this study were reported as mean, standard deviation (SD), and 95% confidence interval (CI). Given the assumption of normality, primary and secondary outcomes were evaluated using the independent samples t-test. The proportion of patients who exhibited disease progression or improvement in Cobb angle between the two groups was compared using the Chi-square test.

All analyses were conducted under a two-tailed hypothesis with an alpha level of 0.05. Statistical analyses were performed using SPSS (version 23.0, SPSS Inc., Chicago, Illinois, USA), and were carried out by a blinded statistician

4.4 The calculation of sample size

The sample size estimation for this study is based on the Cobb angle, which serves as the primary outcome measure. The sample size was calculated using PASS 11.0 In accordance with the principles of clinical superiority trials and preliminary trial results, the clinically meaningful difference in the improvement of the Cobb angle between the DCG and CIG groups is 4, with a standard deviation of 6 for both groups, and a superiority margin of 4 for the Cobb angle. Assuming a power of 80% and a two-sided significance level of 0.05, the estimated sample size is 102 participants (51 per group). Accounting for a 20% dropout rate, a total of 128 participants (64 per group) will need to be enrolled.

4.5 The method of Allocation

Participants were randomly allocated to either the TBEG group or the OBEG group using an online platform (https://www.random.org/). Subsequently, subjects in the OBEG group were assigned the letter "C," while those in the TBEG group were assigned the letter "T." Based on the results generated by the online platform (e.g., C, T, C, T, T, C, …), slips of paper labeled with the letters “T” and “C” were placed in sealed, opaque, and uniformly sized envelopes. After all baseline measurements were completed for the study subjects, the envelopes were sequentially opened to reveal the allocation results. The allocation sequence was prepared by an independent researcher, not involved in the study, using a block randomization model. Given the nature of the intervention, it was not feasible to blind the physical therapists and patients to the allocation results.

5.Exclusion and Inclusion criteria

5.1 Inclusion criteria

The inclusion criteria were as follows:

(a) untreated adolescent males and females with AIS;

(b) AIS patients with a primary curve Cobb angle >10° ;

(c) all subjects aged 9 to 17 years;

(d) AIS patients with a Risser grade ranging from 0 to 4, with female adolescents being within one year post-menarche;

(e) participants capable of understanding and completing complex motor tasks;

(f) patients or their parents proficient in using a smartphone.

5.2 Exclusion criteria :

The exclusion criteria included:

(a) a non-idiopathic etiology of scoliosis determined by clinical information, physical examination, or medical imaging;

(b) AIS patients with other spinal disorders (e.g., tumors);

(c) patients with limb length discrepancy.

6.Research process

6.1 The patients' baseline assessment

(1) Complete basic information such as height, weight, age, and the Rssier grade;

(2) Whether the individuals have disc herniation, nerve compression symptoms, spinal fracture, etc., are determined by X-ray and physical examination;

(3) Baseline Cobb Angle, ATR, and the gait analysis will be assessed.

6.2 Allocation

The patients with AIS will be randomly divided into two groups. The Digital Care group (n=64) underwent PSSE with the help of a telerehabilitation system. The Conventional Intervention group (n=64) will experience PSSE three sessions in the outpatient department of a hospital and complete the home-based PSSE training.

6.3 Intervention

In this trial, HIRS system will be provided free of charge to AIS patients in the DCG group, and physical therapists will encourage timely usage of the HIRS system via

smartphone during the six-month study period. HIRS is a smartphone application available for iOS, Android, and local web solutions. For participants without internet access, hotpots will be provided. The HIRS system consists of three distinct components: the physician's interface, the user's interface, and the data storage module, as depicted in Figure 2. A secure and seamless bidirectional connection is established between the physician's interface and the user's interface, supported by technical experts and IT professionals. The study involves two senior rehabilitation therapists, each with over five years of experience in the diagnosis and conservative management of scoliosis. One of the therapists is a member of SOSORT.

Prior to the commencement of the trial, professional healthcare personnel creates specific PSSE training videos, which, along with detailed instructions, were uploaded to the HIRS system. At the start of the trial, the HIRS system was installed on the smartphones of patients in the DCG group, and personal accounts will be registered via the application. During the initial treatment phase, therapists will transmit digital PSSE training protocols to the patients' personal accounts and provide education on the correct usage of the application. Patients are required to access the application via their personal accounts each time they engaged in PSSE training. Upon initiation of the exercises, patients will follow the instructions provided in the video to complete each movement in the training protocol. If a patient failed to engage in the exercises, the system automatically sends a reminder and notifies the PTs, who then contact the patient to determine the cause of non-compliance.

The PSSE training consisted of daily self-correction exercises and over-correction exercises. The daily self-correction exercises were based on the SEAS method[Error! Reference source not found.,Error! Reference source not found.], where therapists instructed AIS patients in specific three-dimensional corrective postures in the sagittal, coronal, and transverse planes, aiming to realign the spine to a neutral position. To enhance patient self-management of posture, PTs will require AIS patients to maintain the self-corrective posture during routine activities such as sitting, standing, and walking whenever possible. If patients are unable to actively control their posture continuously, they will be encouraged to utilize aids, such as insoles or cushions, to achieve passive correction. Given that the trial participants are students who spend

the majority of their day in a seated position, seated self-correction exercises formed a significant component of the daily training regimen. All AIS patients perform a minimum of 30 minutes of seated self-correction exercises daily for a duration of six months. The over-correction exercises were derived from the Schroth method[11,29,30]. The over-corrective exercise protocols varies according to the PUMCH-SSE classification. During the over-correction training, patients' spines are compelled to maintain a neutral or even over-corrected position. All AIS patients engage in over-correction exercises for 30 minutes per day, at least five days a week, for a duration of six months. The PSSE training regimen, established based on the PUMCH-SSE classification, has been demonstrated to be effective for AIS patients[23, 31].

In this trial, individuals in the DCG group receive a single in-person PSSE training session under the guidance of physical therapists (PTs) in an outpatient setting. During this session, PTs will assist patients or their guardians in installing the HIRS system on their smartphones and provide instructions on how to utilize the system. Simultaneously, individuals with AIS will be required to promptly study educational videos and articles related to scoliosis to enhance their understanding of the condition[Error! Reference source not found.]. Following this session, individuals in the DCG group will complet home-based PSSE training through the HIRS system. The HIRS system automatically records individual PSSE training performance and engagement data, including the number of sessions and duration of training.

In the Conventional Intervention Group (CIG), individuals will receive three 90-minute in-person training sessions per month, under the guidance of PTs, during the first three months, with additional guidance sessions provided as needed. During these sessions, patients or their guardians are required to record the entire PSSE instructional process on video and received printed PSSE exercise materials. Subsequently, patients with scoliosis will complete the six-month PSSE training regimen at home using these videos and materials.

Figure 2. Example of the HIRS system.

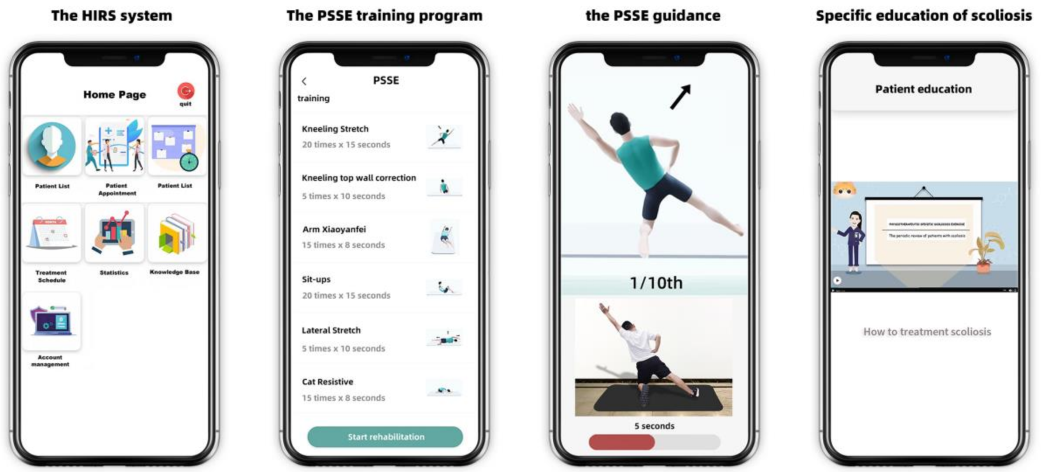

7. The follow-up

All participants will come back to Peking Union Medical College Hospital to complete the follow-up (including measuring the Cobb angle and the ATR, completing the gait analysis and so on) after the 6-month intervention.

8. The Figure of research proccess

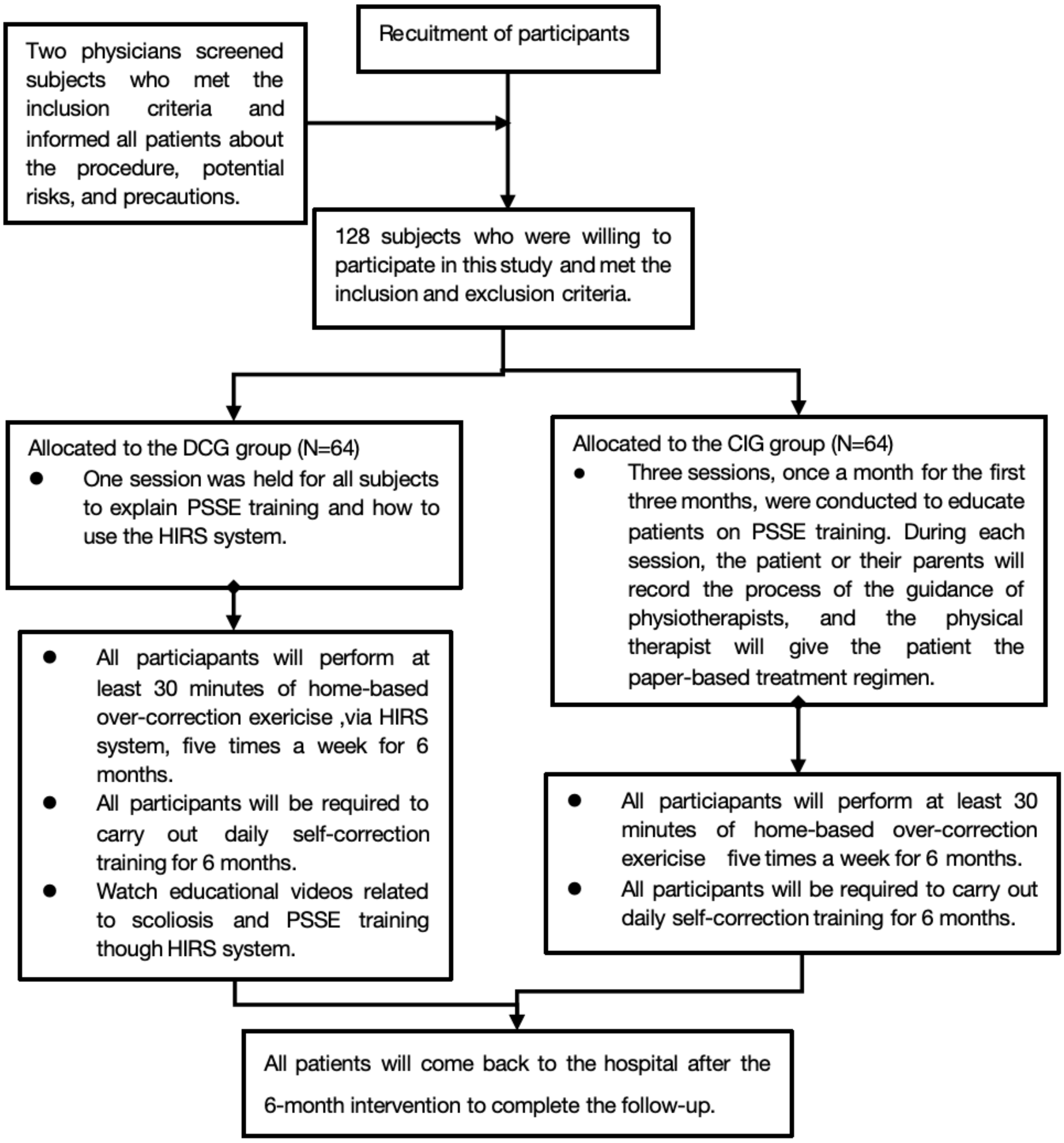

9. Reference

1. Peng, Y., et al. Research progress on the etiology and pathogenesis of adolescent idiopathic scoliosis. Chinese medical journal, **133**, 483 – 493 (2020).
2. Horne, J. P., Flannery, R., & Usman, S. (2014). Adolescent idiopathic scoliosis: diagnosis and management. American family physician, **89**, 193 – 198 (2014).
3. Altaf, F., et al. Adolescent idiopathic scoliosis. BMJ (Clinical research ed.), **346**, f2508 (2013).

4. Yılmaz, H., et al. Prevalence of adolescent idiopathic scoliosis in Turkey: an epidemiological study. The spine journal : official journal of the North American Spine Society, **20**, 947–955 (2020).
5. Trobisch, P., Suess, O., & Schwab, F. Idiopathic scoliosis. Deutsches Arzteblatt international, **107**, 875–884 (2010).
6. Lonstein, J. E., et al. The prediction of curve progression in untreated idiopathic scoliosis during growth. The Journal of bone and joint surgery. American volume, **66**, 1061–1071(1984).
7. Negrini, S., et al. 2016 SOSORT guidelines: orthopaedic and rehabilitation treatment of idiopathic scoliosis during growth. Scoliosis and spinal disorders, **13**, 3 (2018).
8. Negrini, S., et al. 2011 SOSORT guidelines: Orthopaedic and Rehabilitation treatment of idiopathic scoliosis during growth. Scoliosis, **7**, 3 (2012).
9. Gámiz-Bermúdez, F., Obrero-Gaitán, E., Zagalaz-Anula, N., & Lomas-Vega, R. . Corrective exercise-based therapy for adolescent idiopathic scoliosis: Systematic review and meta-analysis. Clinical rehabilitation, **36**, 597–608 (2022).
10. Charalampidis, A., et al. Nighttime Bracing or Exercise in Moderate-Grade Adolescent Idiopathic Scoliosis: A Randomized Clinical Trial. JAMA network open, **7**, e2352492 (2024).
11. Park, J. H., Jeon, H. S., & Park, H. W. (2018). Effects of the Schroth exercise on idiopathic scoliosis: a meta-analysis. European journal of physical and rehabilitation medicine, **54**, 440–449 (2018).
12. Ma, K., Wang, C., Huang, Y., Wang, Y., Li, D., & He, G. The effects of physiotherapeutic scoliosis-specific exercise on idiopathic scoliosis in children and adolescents: a systematic review and meta-analysis. Physiotherapy, **121**, 46–57 (2023).
13. da Silveira, G. E., et al. The Effects of Short- and Long-Term Spinal Brace Use with and without Exercise on Spine, Balance, and Gait in Adolescents with Idiopathic Scoliosis. Medicina (Kaunas, Lithuania), **58**, 1024 (2022).
14. Ceballos-Laita, L., et al. The effectiveness of Schroth method in Cobb angle, quality of life and trunk rotation angle in adolescent idiopathic scoliosis: a systematic review and meta-analysis. European journal of physical and rehabilitation medicine, **59**, 228–236 (2023).

15. Kocaman, H., Bek, N., Kaya, M. H., Büyükturan, B., Yetiş, M., & Büyükturan, Ö. The effectiveness of two different exercise approaches in adolescent idiopathic scoliosis: A single-blind, randomized-controlled trial. PloS one, **16**, e0249492 (2021).
16. Mittermaier, M., Venkatesh, K. P., & Kvedar, J. C. . Digital health technology in clinical trials. NPJ digital medicine, **6**, 88 (2023).
17. Manzak Dursun, A. S., Ozyilmaz, S., Ucgun, H., & Elmadag, N. M.The effect of Pilates-based exercise applied with hybrid telerehabilitation method in children with adolescent idiopathic scoliosis: A randomized clinical trial. European journal of pediatrics, **183**, 759–767 (2024).
18. Pereira, A., Lima, D., Martins, M., Plancha-Silva, T., Amaral-Silva, M., & Marques, E. Idiopathic Scoliosis Trends One Year After COVID-19: A Retrospective Study. Cureus, **14**, e32779(2022).
19. Fan, Y., To, M. K., Kuang, G. M., & Cheung, J. P. Y. . The Relationship Between Compliance of Physiotherapeutic Scoliosis Specific Exercises and Curve Regression With Mild to Moderate Adolescent Idiopathic Scoliosis. Global spine journal, **14**, 447–457 (2024).
20. Simhon, M. E., et al. Completion of a formal physiotherapeutic scoliosis-specific exercise training program for adolescent idiopathic scoliosis increases patient compliance to home exercise programs. Spine deformity, **9**, 691–696 (2021).
21. Liu, D., et al. Effects of Specific Exercise Therapy on Adolescent Patients With Idiopathic Scoliosis: A Prospective Controlled Cohort Study. Spine, **45**, 1039–1046 (2020).
22. Fatoye, F., et al. The Clinical and Cost-Effectiveness of Telerehabilitation for People With Nonspecific Chronic Low Back Pain: Randomized Controlled Trial. JMIR mHealth and uHealth, **8**, e15375 (2020).
23. Yuan, W., et al. A Novel Classification of Juvenile and Adolescent Idiopathic Scoliosis for Conservative Treatment. World neurosurgery, **187**, e447–e452 (2024).
24. Jarchi, D., Pope, J., Lee, T. K. M., Tamjidi, L., Mirzaei, A., & Sanei, S. (2018). A Review on Accelerometry-Based Gait Analysis and Emerging Clinical Applications. IEEE reviews in biomedical engineering, 11, 177–194.

25. Mangone, M., et al. . Gait analysis advancements: rehabilitation value and new perspectives from forensic application. European review for medical and pharmacological sciences, 27, 3 – 12(2023).
26. Resche-Rigon, M., & White, I. R. Multiple imputation by chained equations for systematically and sporadically missing multilevel data. Statistical methods in medical research, **27**, 1634 – 1649 (2018).
27. loom, D. A., Kaplan, D. J., Mojica, E., Strauss, E. J., Gonzalez-Lomas, G., Campbell, K. A., Alaia, M. J., & Jazrawi, L. M. The Minimal Clinically Important Difference: A Review of Clinical Significance. The American journal of sports medicine, 51, 520 – 524 (2023).
28. McGlothlin, A. E., & Lewis, R. J. Minimal clinically important difference: defining what really matters to patients. JAMA, 312, 1342 – 1343 (2014).
29. Büyükturan, Ö., et al. Comparison of the efficacy of Schroth and Lyon exercise treatment techniques in adolescent idiopathic scoliosis: A randomized controlled, assessor and statistician blinded study. Musculoskeletal science & practice, 72, 102952 (2024).
30. Gao, A., et al. Schroth exercises improve health-related quality of life and radiographic parameters in adolescent idiopathic scoliosis patients. Chinese medical journal, 134, 2589 – 2596 (2021).
31. Yuan, W., et al. Differences in Nonspecific Low Back Pain between Young Adult Females with and without Lumbar Scoliosis. Pain research & management, **2019**, 9758273 (2019).
