# Supplementary material for: Digital Physiotherapeutic Scoliosis-Specific Exercises for Adolescent Idiopathic Scoliosis: A Randomized Clinical Trial
Source: JAMA Netw Open. 2025 Feb 18;8(2):e2459929. doi: 10.1001/jamanetworkopen.2024.59929 (PMC11836762; doi:10.1001/jamanetworkopen.2024.59929)
Supplement: Supplement 2. — eTable 1. PUMCH-SSE Classification eTable 2. Changes in Outcomes From Baseline to 6 Months: Per-Protocol Analysis eTable 3. Adjusted Primary and Secondary Posttreatment Outcomes eFigure. Example of the HIRS [file jamanetwopen-e2459929-s002.pdf]

Supplemental Online Content

Yuan W, Shi W, Chen L, et al. Digital physiotherapeutic scoliosis-specific exercises for adolescent idiopathic scoliosis: a randomized clinical trial. *JAMA Netw Open*. 2025;8(2):e2459929. doi:10.1001/jamanetworkopen.2024.59929

eTable 1. PUMCH-SSE Classification
eTable 2. Changes in Outcomes From Baseline to 6 Months: Per-Protocol Analysis
eTable 3. Adjusted Primary and Secondary Posttreatment Outcomes
eFigure. Example of the HIRS

This supplemental material has been provided by the authors to give readers additional information about their work.

© 2025 Yuan W et al. *JAMA Network Open*.

eTable 1. PUMCH-SSE Classification[23]

Type	Subtype	Characteristics of radiograph films
0		Slight curve. Cobb angle 0-10°
I (single curve)	I0	Upper thoracic curve, apex between T1 and T5-T6 intervertebral disc
	Ia	Thoracic curve, apex between T6 and T10
	Ib	Thoracolumbar curve, apex between T10 and T11 intervertebral disc and L1
	Ic	Lumbar curve, apex between L1 and L2 intervertebral disc and L4 and L5 intervertebral disc
II (double curve)	IIa	Upper thoracic curve plus thoracic curve
	IIb	Thoracic curve plus thoracolumbar/lumbar curve
	IIc	Upper thoracic curve plus thoracolumbar/lumbar curve
III (triple curve)		Upper thoracic curve plus thoracic curve plus thoracolumbar/lumbar curve

© 2025 Yuan W et al. *JAMA Network Open*.

eTable 2. Changes in Outcomes From Baseline to 6 Months: Per-Protocol Analysis (N = 111)

Outcomes Variables (median;95%CI)	Digital Care Group (*N*=57)	Conventional Intervention Group (*N*=54)	Estimate the difference between groups	*P*
Cobb angle of the major curve				
Baseline	15.58 (14.49,16.86)	16.30 (15.03, 17.56)	-0.72 (-2.36, 0.92)	.39
6 months	6.16 (4.68, 7.63)	10.89 (9.08, 12.69)	-4.73 (-7.03, -2.44)	<.001
Changes baseline-6 months	-9.42 (-10.65, -8.19)	-5.41 (-6.55, -4.27)	-4.01 (-5.68, -2.35)	<.001
The population of IS patients with progression and improvement of Cobb angle of the major curve				
‘the large improvement’, *N*,(%)	49 (86.0)	29 (53.7)		<.001
‘the mild improvement’, *N*,(%)	5 (8.8)	21 (38.9)		
‘the progression’, *N*,(%)	3 (5.2)	4 (7.4)		
ATR				
Baseline	6.25 (5.58, 6.92)	6.17 (5.15,7.19)	0.08 (-1.11, 1.27)	.90
6 months	5.29 (4.61, 5.97)	5.63 (4.64, 6.62)	-0.34 (-1.52, 0.83)	.83
Changes baseline-6 months	-0.96 (-1.39, -0.53)	-0.54 (-1.03, 0.04)	-0.42 (-1.07, 0.23) •	.64
The pelvic obliquity angle				
The minimum value of the support phase				
Baseline	3.26 (2.62, 3.90)	3.26 (2.62, 3.90)	0.00 (-0.89, 0.89)	.99
6 months	1.50 (1.05, 1.95)	2.74 (2.05, 3.43)	-1.24 (-2.05, -0.44)	.003
Changes baseline-6 months	-1.76 (-2.50, -1.02)	-0.51 (-1.41, 0.38)	-1.25 （-2.39, -0.10)	.03
The maximum value of the support phase				
Baseline	3.08 (2.41, 3.75)	3.30 (2.62, 3.98)	-0.22 (-1.17, 0.72)	.64
6 months	1.09 (0.75, 1.43)	2.07 (1.57, 2.57)	-0.98 (-1.58, -0.38)	.001
Changes baseline-6 months	-1.98 (-2.58, -1.39)	-1.23 (-1.67, -0.78)	-0.76 (-1.50, -0.2)	.05
The minimum value of the swing phase				
Baseline	3.22 (2.55, 3.88)	3.17 (2.51, 3.82)	0.05 (-0.87, 0.97)	.92

© 2025 Yuan W et al. *JAMA Network Open*.

6 months	0.95	1.91	-0.95	.002
	(0.63, 1.28)	(1.38, 2.43)	(-1.56, -0.35)	
Changes baseline-6 months	-2.26	-1.56	-1.00	.03
	(-2.81, -1.71)	(-1.96, -0.56)	(-1.88, -0.12)	
The maximum value of the swing phase				
Baseline	3.33	3.36	-0.03	.95
	(2.65, 4.01)	(2.64, 4.07)	(-1.01, 0.94)	
6 months	0.71	1.58	-0.87	.002
	(0.51, 0.91)	(1.10, 2.07)	(-1.38, -0.36)	
Changes baseline-6 months	-2.68	-1.57	-1.11	.03
	(-3.30, -2.06)	(-2.21, -0.93)	(-1.99, -0.23)	

© 2025 Yuan W et al. *JAMA Network Open*.

eTable 3. Adjusted Primary and Secondary Posttreatment Outcomes (N = 128)

Outcomes (median;95%CI) Variables	Digital Care Group (N=64)	Conventional Intervention Group (N=64)	Estimate the difference between groups	*P*
Primary outcome				
Cobb angle of the major curve	7.55 (6.23, 8.86)	11.78 (10.47, 13.09)	-4.24 (-6.09, -2.38)	<.001
Secondary outcomes				
ATR	5.35 (4.92, 5.77)	5.71 (5.28, 6.13)	-0.36 (-0.96, 0.24)	0.24
The pelvic obliquity angle				
The minimum value of the support phase	1.51 (0.98, 2.03)	2.70 (2.18, 3.22)	-1.20 (-1.94, -0.46)	0.002
The maximum value of the support phase	1.04 (0.74, 1.33)	1.92 (1.63, 2.22)	-0.89 (-1.31, -0.47)	<.001
The minimum value of the swing phase	0.96 (0.57, 1.35)	1.99 (1.61, 2.38)	-1.03 (-1.58, -0.48)	<.001
The maximum value of the swing phase	0.70 (0.39, 1.00)	1.47 (1.17, 1.77)	-0.77 (-1.20, -0.35)	.001

© 2025 Yuan W et al. *JAMA Network Open*.

eFigure. Example of the HIRS

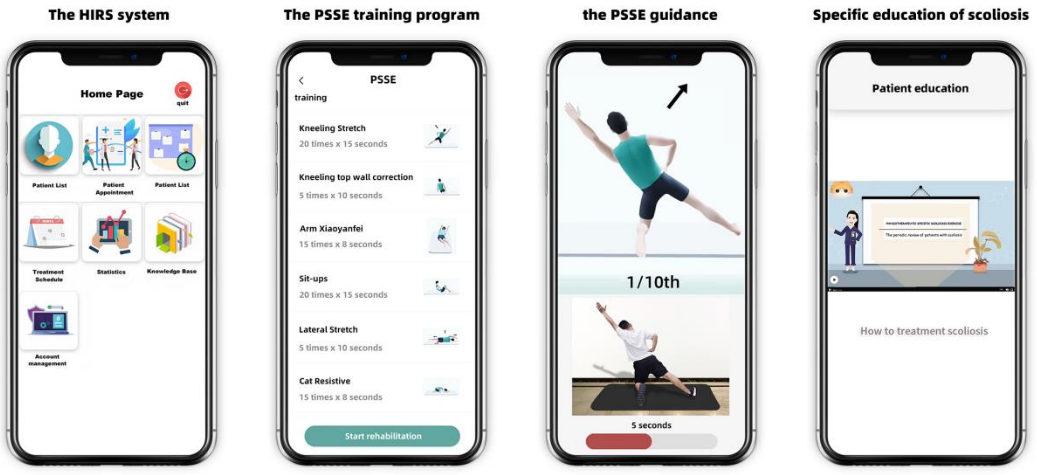

The eFigure was created originally by our research team and has never been published before.

© 2025 Yuan W et al. *JAMA Network Open*.
