# Supplementary material for: Digital Physiotherapeutic Scoliosis-Specific Exercises for Adolescent Idiopathic Scoliosis: A Randomized Clinical Trial
Source: JAMA Netw Open. 2025 Feb 18;8(2):e2459929. doi: 10.1001/jamanetworkopen.2024.59929 (PMC11836762; doi:10.1001/jamanetworkopen.2024.59929)
Supplement: Supplement 3. — Nonauthor Collaborators [file jamanetwopen-e2459929-s003.pdf]

Supplemental Online Content: Nonauthor Collaborators
*First name, last name, and suffix (if applicable) are required and will appear in PubMed.

*Group Name(s): Digital Care Study Group							
***First Name and Middle Initial(s)**	***Last Name**	***Suffix (eg, Jr, III)**	Academic Degrees	Institution	Location (city, state/province, country)	Role or Contribution, eg, chair, principal investigator	Group (if more than 1 Group listed in the byline) and/or Subgroup (eg, Steering Committee)
Wangshu	Yuan		MS	Department of Rehabilitation Medicine, Peking Union Medical College Hospital, Chinese Academy of Medical Sciences and Peking Union Medical College, Beijing, China	Beijing,China	Wangshu Yuan and Weihong Shi contributed equally to this research. The inception of the study,conducting the experimental procedures, the drafting of the manuscript and the data analyses	the Digital Care Study Group
Weihong	Shi		MS	Department of Rehabilitation Medicine, Peking Union Medical College Hospital, Chinese Academy of Medical Sciences and Peking Union Medical College, Beijing, China	Beijing,China	Wangshu Yuan and Weihong Shi contributed equally to this research. The inception of the study,conducting the experimental procedures, the drafting of the manuscript and the data analyses	the Digital Care Study Group

Supplemental Online Content: Nonauthor Collaborators
*First name, last name, and suffix (if applicable) are required and will appear in PubMed.

***First Name and Middle Initial(s)**	***Last Name**	***Suffix (eg, Jr, III)**	Academic Degrees	Institution	Location (city, state/province, country)	Role or Contribution, eg, chair, principal investigator	Group (if more than 1 Group listed in the byline) and/or Subgroup (eg, Steering Committee)
Lixia	Chen		MD	Department of Rehabilitation Medicine, Peking Union Medical College Hospital, Chinese Academy of Medical Sciences and Peking Union Medical College, Beijing, China	Beijing,China	The inception of the study,conducting the experimental procedures, the drafting of the manuscript	the Digital Care Study Group
Di	Liu		MD	Tianjin Binhai Vocational Institute of Automotive Engineering	Beijing,China	conducting the experimental procedures, the drafting of the manuscript	the Digital Care Study Group
Ye	Lin		MD	University of Chicago	Chicago, USA	conducting the experimental procedures, the drafting of the manuscript	the Digital Care Study Group
Qing	Li		MS	Department of Rehabilitation Medicine, Peking Union Medical College Hospital, Chinese Academy of Medical Sciences and Peking Union Medical College	Beijing,China	Substantial contributions to the analysis and preparation of the manuscript	the Digital Care Study Group
Jiandong	Lu		MS	Jiakang Zhongzhi Technology Company	Beijing,China	the analytical process	the Digital Care Study Group
Houqiang	Zhang		MS	Department of Rehabilitation Medicine, Peking Union Medical College Hospital, Chinese Academy of Medical Sciences and Peking Union Medical College	Beijing,China	Substantial contributions to the analysis and preparation of the manuscript	the Digital Care Study Group

Supplemental Online Content: Nonauthor Collaborators
*First name, last name, and suffix (if applicable) are required and will appear in PubMed.

***First Name and Middle Initial(s)**	***Last Name**	***Suffix (eg, Jr, III)**	Academic Degrees	Institution	Location (city, state/province, country)	Role or Contribution, eg, chair, principal investigator	Group (if more than 1 Group listed in the byline) and/or Subgroup (eg, Steering Committee)
Qiyang	Feng		MS	Department of Rehabilitation Medicine, Peking Union Medical College Hospital, Chinese Academy of Medical Sciences and Peking Union Medical College	Beijing,China	the data analyses	the Digital Care Study Group
Huiling	Zhang		MS	Department of Rehabilitation Medicine, Peking Union Medical College Hospital, Chinese Academy of Medical Sciences and Peking Union Medical College	Beijing,China	Substantial contributions to the analysis and preparation of the manuscript	the Digital Care Study Group
