# Supplementary material for: Digital Physiotherapeutic Scoliosis-Specific Exercises for Adolescent Idiopathic Scoliosis: A Randomized Clinical Trial
Source: JAMA Netw Open. 2025 Feb 18;8(2):e2459929. doi: 10.1001/jamanetworkopen.2024.59929 (PMC11836762; doi:10.1001/jamanetworkopen.2024.59929)
Supplement: Supplement 4. — Data Sharing Statement [file jamanetwopen-e2459929-s004.pdf]

Data Sharing Statement

Yuan. Digital Physiotherapeutic Scoliosis-Specific Exercises for Adolescent Idiopathic Scoliosis. *JAMA Netw Open*. Published February 18, 2025.
doi:10.1001/jamanetworkopen.2024.59929

Data
Additional Information: “A prospective, randomized controlled trial to compare the therapeutic effects of Scoliosis specific exercise between remote rehabilitation and outpatient rehabilitation in Adolescent Idiopathic Scoliosis ” &https://www.chictr.org.cn/showproj.html?proj=206129 & CinicalTrial.gov Identifer: ChiCTR2300076563
Data available: No

Additional Information
Explanation for why data not available: contact the corresponding author
